# The Transformational Experience of Junior Nurses Resulting from Providing Care to COVID-19 Patients: From Facing Hurdles to Achieving Psychological Growth

**DOI:** 10.3390/ijerph18147383

**Published:** 2021-07-10

**Authors:** Yuk-Chiu Yip, Ka-Huen Yip, Wai-King Tsui

**Affiliations:** School of Health Sciences, Caritas Institute of Higher Education, Hong Kong, China; khyip@cihe.edu.hk (K.-H.Y.); ztsui@cihe.edu.hk (W.-K.T.)

**Keywords:** coronavirus disease 2019 (COVID-19), junior nurses, transformational experience

## Abstract

The rapid spread of coronavirus disease 2019 (COVID-19) has put significant pressure on junior nursing staff. The objective of this study was to examine the in-depth experiences of junior nurses in providing care for COVID-19 patients within an acute care setting. This study employed a phenomenological method to understand the situation from a first-person perspective. Purposive sampling was used. Interviews were performed with 40 junior nurses (<4 years of clinical experience) who provided direct care to COVID-19 patients in isolation wards in acute care settings in Hong Kong. The interviews were conducted from 1 January 2021 to 24 May 2021 via virtual conferencing software (Zoom) to maintain social distancing, and the responses were analysed using Colaizzi’s seven-step method. Junior nurses’ psychological experiences of providing direct care to COVID-19 patients were categorised into four main themes. First, there were hurdles in the early stages, in which participants experienced negative emotions, such as fear, anxiety, helplessness, and fatigue. Somatic symptoms, such as headaches and sleep disturbance, were reported. Second, the adoption of self-care coping strategies enabled nurses to confront the hurdles, signifying the start of self-transformation. Third, junior nurses maintained positivity under pressure by appreciating their sources of support (including their families and other important relationships in their lives). Professionalism was also found to reinforce positivity. Fourth, self-transformation resulted in psychological growth, which prepared junior nurses to be resilient and confident in their clinical practice to take up future challenges in the ongoing battle against the pandemic. The hurdles experienced by junior nurses at the early stage of their work in isolation wards provided the foundation upon which self-transformation took place. Being able to employ self-care coping strategies and further sustain positivity characterised the self-transformation process. Eventually, junior nurses became resilient and more capable of understanding both the negativity and positivity of their experiences. The self-transformation process also enabled junior nurses to recognise and appreciate the wider support system from various parties in society.

## 1. Introduction

Coronavirus disease 2019 (COVID-19) is caused by a novel variant of the infectious coronavirus that was initially recognised in Wuhan, China, on 31 December 2019 [1]. The disease was classified as a pandemic by the World Health Organisation (WHO) on 11 March 2020, because of the rapid global spread of the virus [2]. Approximately 20% of COVID-19 patients suffer serious complications that require oxygen therapy or other forms of in-hospital intervention, and 5% of patients need to be admitted to intensive care [3].

Nurses have an important role to play in the support and assistance of patients during in-patient care. However, the nurses themselves are at considerable risk of contracting the virus, and it is virtually impossible to eliminate this threat [4]. The virus is highly contagious and can threaten the lives of nurses, and as it is a novel variant, the information related to standards of care and infection control is continually evolving. The influx of infected patients has led to significant alterations to the care settings in which nurses work, thus creating considerable challenges for nurses. Nurses are instrumental and highly involved in efforts to combat the virus. Providing 24/7 nursing care means that nurses are in proximity to COVID-19 patients from their admission to their discharge. Among healthcare professionals, nurses are most exposed to psychologically distressing situations during these difficult times [5,6].

Previous research has shown that, when unexpected natural disasters or pandemics arise, nurses frequently disregard their own health and safety to address the situation in front of them, working selflessly due to their strong ethics and professionalism [7]. Simultaneously, nurses may feel physically and mentally stressed, experiencing isolation and helplessness when faced with dangers to their health and the high-pressure continuous work required when public health emergency situations arise [8]. Research has demonstrated that while working closely with patients suffering from novel infectious diseases, nurses frequently experience fatigue, fear, anxiety, sleep disorders, loneliness and other challenges to both their physical and mental health [9,10,11,12,13,14]. One study showed that nurses attending to SARS patients experienced insomnia (37%), depression (38.5%), and posttraumatic stress (33%) [14]. However, some studies have also shown that working on a collective initiative to combat an epidemic requires nurses to grow professionally in the process and develop resilience to overcome the challenges posed by the outbreak [10,15].

Given that the COVID-19 pandemic is caused by a novel virus, many nurses are thus called to the frontline to provide care to patients, and these nurses may not be familiar with the disease. At the time this research was undertaken, junior nurses with fewer than 4 years of clinical practice were recruited on a voluntary basis to offer their professional help in acute care settings. This group of nurses has been under-researched in terms of their unique experiences on the frontline of the battle against the disease. Current research has noted that physicians experienced severe psychological distress [16,17]; however, there is little research regarding the psychological experiences faced by junior nurses who have been practicing for a certain period “in the field”—providing care to patients with confirmed COVID-19 in acute care settings. To fill this knowledge gap, this qualitative research investigated the in-depth and subjective experiences of junior nurses who provided direct care to patients with confirmed COVID-19 for at least six months using semi-structured interviews and employing a phenomenological methodology [18].

This research has practical implications in that the psychological conditions potentially affecting junior nurses, including fear, anxiety, and burnout, could be shown with empirical evidence so that timely and appropriate support could be provided by healthcare management who are responsible for developing policies to safeguard the mental health of nursing staff [19,20].

## 2. Methods

### 2.1. Research Design

In this qualitative study, junior nurses were comprehensively interviewed about their psychological experiences providing direct hospitalised care for patients with confirmed COVID-19. The data were analysed based on the phenomenological methodology suggested by Colaizzi [18]. With this method, the researchers attempted to understand the participants’ subjective feelings and experiences by mentally returning to the situation itself.

### 2.2. Participants

The participants in this study were 40 junior nurses attending to patients with confirmed COVID-19 at public hospitals in Hong Kong, and purposive sampling was used. To recruit these participants, our research team approached four hospital clusters where isolation wards were established to admit patients with confirmed COVID-19 for hospitalised care. The research team members informed the department operation managers of these clusters information about the study and expressed our interests in interviewing their junior nursing staff. The research team members then individually followed up with junior nurses who obtained our research team contact information (email and/or phone) through their supervisors and showed interest in participating in this study. The inclusion criteria included the following: (1) being a nurse with at least 6 months (continuous or intermittent) of clinical experience in providing direct care to patients with confirmed COVID-19 in an isolation ward, (2) volunteering to participate in the study, and (3) being a nurse with fewer than 4 years of practical experience after graduation from pre-licensure academic studies. In this study, isolation wards were defined as wards in acute care settings that were equipped with multiple airborne infection isolation rooms (AIIRs) for in-patients with confirmed COVID-19. Each AIIR was a single-occupancy negative-pressure room with at least 12 air changes per hour. In addition, the exhaust of air from each AIIR within the isolation wards had to pass through a high-efficiency particulate air filter. Within each AIIR, an en-suite toilet facility was provided for infection-control purposes, such that individually isolated patients within an isolation ward did not share a toilet. Individual interviews continued until data saturation was reached at the 37th interview, after which no new information arose in the next three individual interviews (with two female participants and one male participant). The participants’ characteristics can be found in Table 1. We enrolled six male and 34 female nurses between the ages of 21 and 35, with an average age of 28. The participants had an average of 7.5 months of experience in providing care to patients with confirmed COVID-19. At the time of the interview, the participants were 24–48 months (mean, 39 months) post-graduation. Among the participants, 38 nurses possessed a bachelor’s degree, and two nurses had graduated with a higher diploma. Regarding marriage and children, one nurse was married with children, one was married without children and 38 were unmarried and without children.

### 2.3. Data Collection

All interviews were conducted by the researcher (Y.C.) at the participant’s preferred time in a private password-protected Zoom (Nasdaq, San Jose, California, U.S.) meeting room from 1 January 2021 to 24 May 2021. Each interview took about 60–120 min, and the same interview guide was always used to reduce variation in the data-collection process. A panel of two experts—an academic qualitative scholar and a psychological consulting specialist—was formed to review and affirm the validity of the interview questions. All interviews were audio-recorded with the participants’ written permission. Field notes were taken during the interviews to facilitate the collection of contextual information and thus, the data analysis. The interview guide questions are listed in Table 2. If the participant exhibited emotional distress during the interview, adequate psychological support was provided to prevent further psychological harm. The participants were also informed that they could withdraw consent at any time.

### 2.4. Data Analysis and Rigor

Data analysis was performed immediately after data collection using NVivo software (NVivo Version 12, QSR International). For individual interviews, the interview tapes were transcribed verbatim by two researchers (Y.C. and K.H.). Colaizzi’s [18] phenomenological analysis method was used for systematically organising the data and field notes. This method includes a seven-step process to reveal emergent themes through (1) familiarisation with the collected data, (2) identification of the significant statements, (3) formulation of meanings alongside the use of the researcher’s reflective bracketing, (4) clustering of themes, (5) formation of an exhaustion description, (6) development of the fundamental structure of the phenomenon, and (7) verification of the exhaustive description and fundamental structure [18]. 

Specifically, the recorded data of each interview were transcribed word for word and analysed by Colaizzi’s [18] phenomenological analysis method throughout the data-analysis process. Two members of the research team (Y.C. and K.H.) independently reviewed the interview materials, summarised and extracted meaningful statements, and formulated different themes. The researchers compared the subthemes and identified the differences and similarities between them by looking at the picture holistically. The study’s authors (Y.C., K.H., W.K.) discussed the results of their analyses to reach a consensus. To ensure a high level of rigor in this study, the study referenced the stringent criteria established by Lincoln and Guba [21]. The summary table provided below (see Table 3) illustrates how each of the criteria, in relation to credibility, dependability, confirmability, and transferability, was fulfilled. Member checking was also performed.

### 2.5. Ethical Review

All participants were informed of the details of the study. Informed consent was obtained from all participants. All methods were carried out in accordance with the relevant guidelines and regulations of the authors’ affiliated institution. Participation was voluntary, and all participants were informed that they could withdraw from the study without any consequences. The confidentiality and anonymity of the participants were strictly ensured with encryption. Only the researchers had access to the study data. Ethical approval was obtained from the Research and Ethics Committee of the Caritas Institute of Higher Education in Hong Kong (HRE200133). 

## 3. Results

Using phenomenological methods, we explored the psychological experiences of junior nurses providing direct care to patients with confirmed COVID-19. Four themes were identified from the observations, and they are summarised below:

### 3.1. Theme 1: Hurdles in the Early Stage

When participants experienced their first working week in isolation wards, they all demonstrated high stress and negative emotions.

#### 3.1.1. Suffering Due to Overwork and Burnout

The increasing intake of confirmed cases of COVID-19 patients came with an increase in workload that was substantially greater than the normal workload experienced in nursing care services. They all complained that they were feeling unwell and tired. They felt exhausted both physiologically and psychologically.

“After working non-stop for 8 h a day and wearing personal protective equipment all day, I often suffered from headaches and chest tightness. At the same time, the band of the surgical mask on my ears made me uncomfortable. Every time I finished my shift and took off my PPE, I was sweaty and my entire body ached. I felt very tired as if I was about to collapse, and I could fall asleep easily at any time. I often wished there was a bed right in front of me so I could jump onto it and fall asleep right away.”

“I was under a lot of pressure [said in a slightly raised tone while leaning towards the interviewer] … I was mentally exhausted from keeping abreast of the updates on the admission situation in the various isolation wards … The rapidly increasing number of protocols relating to isolation and disinfection and the overwhelming number of updates on nursing and medical interventions simply wore me down … I could not afford the time to have a psychological consultation, as I did not even have enough sleep.”

Participants also expressed feelings of stress and helplessness in the face of a limited supply of personal protective equipment (PPE). They felt frustrated trying to perform nursing procedures when resources were tight. Meanwhile, the inadequate stock of PPE posed huge challenges to the nursing staff because they had to strike a delicate balance between meeting patients’ needs and controlling the consumption of PPE in their daily practice within the boundaries set by the hospital infection-control protocols. Most participants adopted radical measures to minimise the usage of PPE as much as they could.

“I felt drained and somewhat less capable to deliver care when resources were unprecedentedly and extremely tight … I decided to drink less water and eat much less to reduce both the frequency of going to the toilet and, hence, the need to change my PPE.”

#### 3.1.2. Fear and Anxiety 

Participants expressed fear and anxiety when they were asked by their department if they were willing to work voluntarily in isolation wards, because most of them had been practicing mainly in general adult medical and/or surgical wards in the past few years. Specifically, participants doubted if they could cooperate well at a professional level with the physicians and nursing staff in isolation wards because some of them were unfamiliar with the nursing care workflow and the operational logistics of isolation wards, both of which were different from those of general adult wards.

“I worried that my nursing colleagues would feel unhappy with me because I worked slower than them … I was afraid of potential criticism during my adaptation period [said with a frown] … I worried that the doctors would blame me because I was considered not as competent as others who were used to working in an isolation ward setting … I could not sleep very well and I had an upset stomach once I knew I would soon be relocated.”

“I used to work in paediatric units and adult medical wards … The nursing and treatment items there (isolation wards) were all placed in different locations from the general ward settings … The operating procedures and daily routines of nursing care in an isolation ward were different … I felt very anxious and had difficulty falling asleep … I pushed myself to the limit to adapt to the ward practices as quickly as I could.”

Participants also expressed that their fear and anxiety stemmed from their limited knowledge of the disease and the relevant strategies of nursing management (for example, the mechanisms of pathogenesis, possible routes of transmission, treatment algorithms, and nursing interventions). They considered the above-mentioned knowledge important for them to accurately evaluate a patient’s condition and treatment progress and to plan their nursing care accordingly.

“Honestly, the feeling of fear drove me to put extra effort into learning … My colleagues and I did not want to appear petrified when patients’ inquiries and needs arose … I would have felt uneasy if I were less knowledgeable than my co-workers and senior nurses who worked diligently to serve the patients with professionalism.”

“I was particularly concerned about possible sudden complications, like shortness of breath or unknown psychological conditions … I am very concerned about the mental well-being of patients who need to receive treatment in an isolation ward. I need more knowledge from both research and updated nursing guidelines to support my practice. I felt dreadful for my lack of preparedness [sighed deeply].”

“I always worried about what I should do if the patient’s condition deteriorated in a split second … I wondered whether the resuscitation effort would be uncoordinated because the nursing team was newly formed.”

Social media (for example, disease updates forwarded through Twitter) may also have increased the fear and anxiety of junior nurses, as social-media messages may have suggested a seemingly “real” and particularly high risk of mortality for the individuals who contracted the novel virus.

“I was academically trained to appraise scientific evidence … I believe in science … Yet I could not distance myself from the news that naturally came up on my Facebook and Twitter pages … These newsfeeds about patients with confirmed COVID-19 terrified me because they focused on reporting the death rates and how critical the patients were.”

#### 3.1.3. Two-Way Relationships between Family Members

All participants came from families with spouses or aged parents. They cared greatly about the health of their family members and were worried about transmitting the virus to them if they got infected. Some participants had not informed their families that they had begun working in an isolation ward. Because of the nature of the disease (i.e., infected individuals may be clinically asymptomatic), many of the participants decided to stay away from their parents and spouses to protect their health and, in the meantime, to prevent any transmission of the virus from the hospital to the community.

“I did not want to emotionally burden my husband during the pandemic. I did not let him know that I was working in an isolation ward … I applied for a subsidy from my hospital so that I could rent an apartment … My husband always cares about me, and I believe I have a duty as a responsible wife to protect him.”

“I dared not tell my parents that I was working in an isolation ward. Every day, I called my parents after work and told them I would stay and rest in the dormitory instead of returning home. I mostly stayed in a hotel when I was off duty… I did not want to spread the virus to my family or the community … Still, I was very worried about my dad, mom and grandma during the pandemic [tears up], and I knew they were worried about me too.”

While participants demonstrated commitment to their professional duties in isolation wards, they were cognizant of the concern and worries from their families. The fact that their nursing work involved physical care and a risk of contracting the novel virus was a major disquieting factor for their family members. This reciprocal pattern of concern (both from the families to the participants and vice versa) was evident, as shown by the following quotes.

“My mother was very worried that I would get infected by performing suction for patients … She texted me a lot to check if I was okay at work. She had never been so anxious, even though she knew all about my profession and my current working environment (in an isolation ward) … Yet I did not want my mother to be so uptight about my health; it was not good for her mentally.”

“My mum kept an eye out for news of any healthcare workers contracting the novel coronavirus. She looked so worried that I might get infected. That is why I was equally worried about her … I knew she was mindful (more than ever) of the relevant news because I worked so closely with patients.”

### 3.2. Theme 2: Self-Care Coping Strategies

Participants relied on their own self-care coping methods to manage the unprecedented work challenges that arose frequently in their clinical environment, as well as their emotions (including ups and downs) during the pandemic. 

#### 3.2.1. Continual Adjustment in Life

All participants engaged in their own psychological self-defence mechanisms, including distraction, humour, and rationalisation. Many participants channelled their emotions by confiding in trusted colleagues and companions. They relieved their stress and addressed their grievances either directly or indirectly through psychological tactics, online media, or other activities (for example, diary-writing, rhythmic breathing, meditation, and listening to music).

“I rationalised things so I felt better while stressed. It was easy. When I got stressed for all sorts of things like criticism from colleagues, I told myself I was not that bad … I may have been junior (in terms of practicing experience), and I had inadequate knowledge … But I could improve by some self-learning and things would be fine.”

“I wrote a diary on Instagram to share my excitement and sadness … Sometimes, I liked having video calls with my boyfriend to seek consolation. He is good at telling me humorous stories … I also felt less anxious when I practiced rhythmic breathing or distracted myself by listening to music.”

Some participants increased their sleeping time by going to bed earlier when they were under immense pressure. Some changed their diet and began exercising regularly to maintain their mental health.

“I thought sleeping more is the best way to reduce stress. It temporarily took the emotional burden and stress off my shoulders … I also liked doing some simple exercises like stretching and yoga regularly. Exercise brings me relaxation.”

“I was more aware of the importance of self-care to my well-being during this pandemic. For example, to strengthen my immune system, I was more selective in my diet choices. I ate more healthy food for breakfast, lunch and dinner.”

#### 3.2.2. Feelings of Unity

Participants pointed out that there could be various sources of stress and anxiety in one’s practice in an isolation ward. Nonetheless, having a feeling of unity with the nursing team represented an effective coping strategy for many participants. Participants further elaborated that, because they felt the solidarity and unity of a nursing team, they were more active in seeking help for their negative emotions and difficulties. On many occasions, these negative emotions and difficulties could be mitigated through mutual support and motivation from colleagues.

“Although I was new to the (isolation) ward, my feeling of unity drove me to seek clarification when I was in doubt … When miscommunication was largely prevented by my active efforts, my anxiety decreased with time.”

“I thought my adaptation time was shorter than others because, at the start of my work in this unfamiliar ward, I regarded myself as an integral part of the team … I supported others, and others did the same for me … You must actively unite with your colleagues to manage the psychological hurdles arising from this rapidly changing pandemic situation [said in a firm tone of voice].”

#### 3.2.3. Anchoring and Holding On

Participants felt uncertain about how to manage the hurdles and negative emotions, such as fear and anxiety, that arose from their practice. The volatility of the pandemic and the unclear implications of the nursing practice increased the participants’ desire to search for an anchor point. The anchor point provided participants with psychological comfort and reassurance for them to cope with the hurdles. Participants identified this anchor point by actively reviewing the literature, international reports and any useful information on the Internet to compare what they encountered with the experiences around the world.

“I needed a reference point psychologically. I was eager to know how the hardships I encountered in clinical settings were managed in other countries … I felt more assured of my nursing practice if I knew other (developed countries) did similarly.”

“I looked over international materials to make sure I was not alone. I thought finding an anchor point in my mind was a self-care method in this difficult time.”

Participants were able to hold on, continuing their hard work in clinical areas, when they derived a certain level of mental calmness from the psychological anchor point. They expressed that identifying an anchor point represented a rational way to manage the hurdles.

“When I felt distressed, I looked around at what was happening throughout the world. I motivated myself by thinking up some reasons that helped me make sense of the clinical impacts of this pandemic.”

### 3.3. Theme 3: Staying Positive under Pressure

Participants stayed positive under pressure by appreciating their sources of support, including their families, colleagues, friends, and other important relationships in their lives. Professionalism was found to play a role in reinforcing positivity. Participants were eventually able to sustain the positivity and, through self-transformation, summon the courage to face the stress and future challenges in their practice.

#### 3.3.1. Appreciation for the People You Love and Live with

Participants expressed that working in an isolation ward and providing direct care to patients with COVID-19 represented a unique (albeit stressful) experience in their nursing practice. Participants admitted that they had not “lived through” any pandemic as they had at this time, because most of them were children during historical pandemics. For example, participants recalled that they were still in primary education when the severe acute respiratory syndrome—commonly known as SARS—pandemic struck Hong Kong in 2003. As the participants suggested, the uniqueness of the experience was characterised by being at the frontline in the battle against this pandemic, being a member of a nursing team to provide professional care, and being grown up and thus, accountable to their families. By reflecting upon the gain from this unique experience, participants were able to stay positive under stress.

“My lovely parents supported and encouraged me to contribute in an isolation ward … I was so thankful for all my loved ones because they rekindled my inner spirit as a nurse … I am blessed and indebted to them for their care, love, motivation and support.”

Participants also derived positive energy by reflecting upon the valuable relationships in their lives. They appreciated the essential affection, love, and friendship in their lives. They felt more positive because of an increasing awareness of the established rapport between the persons they knew and worked with and the emotional attachment to their families.

“The working experience in this pandemic made me feel that nothing (people and relationships) should be taken for granted in life … My mum always motivated me and gave me confidence … I felt I am living in an enabling living and working space … my helpful colleagues gave me a lot of strength when I was stressed.”

#### 3.3.2. Professional Identity and Sense of Responsibility

Participants believed that their professionalism kept them motivated to engage in the fight against the COVID-19 pandemic. Most of them lifted their spirits by reminding themselves of the fundamental nature of their nursing duty and their invaluable role in this fight. Their professionalism strengthened their positive belief regarding the need to perform and undertake duties of the nursing profession.

“I kept reminding myself that this is my duty as a nurse … I was involved in lifesaving work every day … I was trained to provide high-quality and holistic care that aims to alleviate patients’ suffering.”

“Although I was scared about the pandemic, I did not back off … I considered it my responsibility to take good care of the patients. This is what I believe, and I must go on … I think I was internally driven to work based on my philosophy of nursing.”

#### 3.3.3. Self-Transformation: From Fear to Courage

The participants achieved self-transformation when they went through the process of initially being stressed, afraid, and anxious to then staying strong through psychological adjustment. They eventually moved towards positivity by building courage with the adoption of self-care coping strategies, sustained their psychological growth, and became more prepared for the tasks ahead in their clinical practice.

“At that moment, I felt that there was nothing I could not overcome … I had become one of the essential members of the nursing team [laughing out loud] … I was not scared anymore. What else could put me off now that I was not even afraid of death?”

“After all these days, I never thought that I could be so strong [said with a sense of pride and a bright smile] and determined to take up the challenge.”

### 3.4. Theme 4: Perceived Negativity and Positivity: Two Sides of Emotions

Although participants had negative feelings, like fear, anxiety, and worries, at the start of their work in isolation wards, being able to confront the hurdles signified the beginning of their self-transformation to become a resilient nurse in the battle against the pandemic. Through the self-care coping processes, they stayed positive and were more prepared for the future stressful situations in their practice. Ultimately, they were more capable of understanding both the negativity and positivity of their experiences. Participants emphasised that gaining confidence from practicing “in the field” is an essential factor in achieving self-transformation and subsequently recognised and appreciated the wider support system from various parties in society.

#### 3.4.1. Gaining Confidence

All participants gained confidence with time by working in the field and resolving the emerging difficulties with courage. In taking up challenges, they were forced to learn and adapt to the changes brought about by the pandemic. Although they experienced anxiety and fear initially, these feelings decreased with an increasing level of confidence. The confidence gained from practice contributed to the resilience of the participants, signifying an important outcome of self-transformation. While participants began to gain confidence to play an active role in dealing with the effects of the pandemic at their workplace, they acquired a greater awareness of and increased appreciation for the effects of the wider policies relating to the control of the pandemic. They also demonstrated more determination to continue contributing in their professional roles.

“I knew the government had been working hard, but at the beginning, I did not realise the effects … I tended to perceive their work negatively… But now, I think I have grown a lot in the past half year … Lately, I was so excited to hear that Lei Yue Mun Holiday Camp and Asia World-Expo had been converted into provisional hospitals to cope with the increasing number of patients with COVID-19. This played a significant triage function in our battle.”

“Being in the field was a vital learning process, even though the psychological growth I attained did not come easy … After all, a resilient nurse cannot be nurtured by learning within the school’s environment. To me, building confidence in myself and persevering in a time of stress made me succeed in this journey.”

#### 3.4.2. Sources of Support from Various Parties in Society

As opposed to what they perceived at the beginning of their work in isolation wards, participants who had undergone self-transformation understood that, without concerted efforts from society, a single profession could not succeed in winning the battle against this pandemic. Following the self-transformation, they demonstrated maturity in terms of how they perceived the roles of their profession and the stakeholders in society. For example, they were grateful for the cooperation and respect from the patients.

“Those outside the hospitals claimed that nurses were afraid of being infected with COVID-19, but it was not like that. I had never thought about quitting the job. I felt safe with the (infection control) protocols and felt supported by the hospital … I felt cheerful when the patients thanked us for our care.”

Participants also appreciated the support from the hospital management, particularly the welfare provisions that were encouraging and supporting the nursing staff. The support from senior colleagues gave the participants hope. Other forms of support from society were equally valuable to the participants.

“The hospital provided us (nurses) with extra cash allowances and hotel accommodation allowances. After 2 weeks of nursing work, we (nurses) were given an extra day of paid leave so that we could have ample rest … From time to time, some companies donated small gifts and supplies to support us in this fight against the pandemic. I am very moved [smiles]! Even our professional nursing bodies (i.e., the Association of Hong Kong Nursing Staff and the Hong Kong Academy of Nursing) have sent us gifts to show their support. Many video clips with supportive and encouraging messages to the nursing staff have also been uploaded online.”

## 4. Discussion

This research employed phenomenological methods to examine the experiences of junior nurses who provided direct care for patients with confirmed COVID-19. The outcomes of the interviews could be broadly categorised into four themes: (1) hurdles in the early stage, (2) self-care coping strategies, (3) staying positive under pressure, and (4) perceived negativity and positivity: two sides of emotions. 

The interviews revealed that the participants experienced significant discomfort and exhaustion as a result of their heavy workload, the scale of the outbreak, the large number of patients who required care and treatment, and the insufficient supply of PPE. These findings are consistent with those from studies on the outbreaks of MERS-CoV and Ebola [13,22]. 

In the current study, junior nurses expressed worries about their family members, corroborating a previous study by Lee et al. [23]. These emotions were prevalent among nurses who had elderly individuals or children in their families. The nurses interviewed during this research described how they had experienced strong negative emotions, such as anxiety, fear, and helplessness. Such emotions have been described in several previous studies and can be attributed to a lack of knowledge and experience, a sense of psychological helplessness, health and safety concerns, and fatigue [11,24]. 

The findings of this study revealed that the negative emotions encountered by the junior nurses were stronger in the first week of their clinical practice in isolation wards. Junior nurses may be provided with opportunities to undergo voluntary stress assessments as soon as they undertake pandemic prevention tasks in the initial few weeks or after a certain number of months. Healthcare management may consider supporting junior nurses throughout their adaptation process with specialised, adaptable, and continued interventions to encourage emotional release and to safeguard their mental health [13,23,25]. Solid support systems, such as sufficient PPE, training, and team support, may also be made available to help junior nurses adapt to the demands of providing holistic care to patients during a pandemic.

The participants in the current study revealed that they used several psychological strategies, such as distraction, awareness of the importance of self-care, and rationalisation, to deal with the challenges they encountered. Previous studies have reported that the application of coping strategies can assist clinicians in dealing with stress and in maintaining positive mental health when encountering an epidemic [26]. This study also found that junior nurses deal with stress through processes such as employing self-care coping strategies and seeking social support. Our results support Mak, Law, Woo, Cheung, and Lee’s [27] finding that nurses’ ability to adapt on a psychological level and their access to social support play important roles in their ability to undergo psychological adaptation and self-transformation when encountering outbreak stress. When acting under stress and pressure, nurses may adopt psychological self-modifications to gain confidence and build courage and resilience for emerging challenges in their clinical practice.

The interviewees in this study described how they used various methods to reduce stress, including listening to music, diary-writing, and engaging in breathing exercises. This finding corroborates previous studies on how nurses attending to patients in SARS wards employed various approaches to manage stress and pressure [23,28]. Furthermore, we found that the nurses involved in our study exhibited a strong sense of team solidarity that enabled them to cope with the hurdles at the early stage of their work in isolation wards, echoing the findings of Kim [11] and Shih, Liao, Chan, Duh, and Gau [29]. On a holistic level, junior nurses tended to modify their cognitive rationality to adjust to the demands presented by the pandemic. This could be attributed to the fact that healthcare practitioners possess strong healthcare knowledge and understanding and have a more logical and optimistic mindset [30]. Per the stress and coping model presented by the American psychologist Richard Lazarus, the extent to which stressors are effective is directly correlated with the process by which one engages in cognitive appraisal and the coping strategies involved. When operating in high-pressure situations, junior nurses continually modify their cognitive appraisal by applying their professional knowledge to seek support from members of their team, to encourage self-psychological harmony, to perform altruistic acts, and to take active steps to decrease stress and modify their nutrition, exercise, and sleep to respond to changes in the internal and external context and avoid stress-related impacts on their health and ability to perform their nursing duties [10,25].

Previous research has found that epidemics may cause significant psychological trauma to healthcare workers [16,17]. However, the outcomes of this study revealed that junior nurses developed on a psychological level when under stress. Through self-transformation, junior nurses engaged in a process of self-evaluation and ultimately responded positively—for example, by voicing their appreciation for their families and social support networks. The perception of accountability associated with professional ethics during an epidemic inspired nurses to enthusiastically contribute to anti-epidemic tasks and enhanced their sense of professional pride and identity [7,27]. These findings agree with those of a previous study [15]. Proactively supporting junior nurses to achieve psychological development during a pandemic could facilitate their psychological adjustment.

In the current study, we found that junior nurses experienced many positive emotions, such as confidence, courage, and gratitude, when providing care to patients during the pandemic. These positive emotions characterise the process of self-transformation. This finding contrasts sharply with previous research that was limited to discussing the negative emotions nurses encounter due to outbreak stress [16,17]. However, some studies have described comparable findings to ours [10,15,16]. Researchers have found that positive emotions can play a significant role in people’s ability to adjust or respond to psychological trauma [31]. Positivity can have protective implications in terms of psychological trauma during tragedies and can stimulate psychological recovery from posttraumatic stress [32]. Thus, psychological support services that could be provided to junior nurses during a pandemic may include offering strong social support, encouraging positive coping styles, and promoting positive emotions.

During an outbreak, early training and confidence in skills and safety represent fundamental attributes that support nurses’ willingness to engage in anti-epidemic work [33]. Mental and physical rewards also represent significant supportive elements [24]. The junior nurses who participated in the current study typically believed that their positive emotions were attributable to the multi-faceted support they received from family, friends, patients, social groups, team members, and the organisations in which they worked. Similar studies that support our findings can be identified in the literature [14,15,16]. 

### 4.1. Strengths

In this study, the data related to the experiences of junior nurses who provided direct care to patients with confirmed COVID-19 were compiled over nearly 6 months via a series of interviews. This helped us gain insights into the lived experiences of junior nurses, who remained an under-researched group at the time this research was undertaken. The methodology of this study enabled us to collate in-depth and reliable information that can allow other researchers to interpret the findings within a clearly described context. Unlike previous studies, our study revealed the process of self-transformation through which junior nurses developed self-care coping strategies in the face of pressure and ways to maintain positivity, confidence, and the ability to appreciate the wider support system in society. These attributes helped junior nurses to become resilient. 

### 4.2. Limitations

First, as this study took the form of qualitative research, it involved a limited sample size. The sample in this study may be biased towards participants who had strong family and/or social support, and as reflected in the results, these participants were able to overcome some of the stress in their nursing work. This limitation could be addressed in future studies by enrolling junior nurses who may not have strong family or social support. For example, participants’ perceived social support could be measured using an established and validated instrument before their interviews. Based on the measurements, researchers could further analyse and interpret the collected data, providing new perspectives on how varying levels of perceived family and/or social support may alter the coping processes of junior nurses in times of stress in their caregiving work during the COVID-19 pandemic. Caution should therefore be exercised in interpreting the results of this study, since junior nurses who do not have strong family and/or social support may have unique emotional and psychosocial needs arising from their practice in the pandemic as well as their difficulties in coping with the stress originating from their caring responsibilities at work. Second, the participants were limited to Hong Kong-based junior nurses who had recently completed their pre-licensure training. Practicing nurses who had undergone pre-licensure training abroad before returning to work in Hong Kong were not included in this study due to their much smaller proportion in public hospitals in Hong Kong. Third, because of the risks associated with a pandemic and the need to avoid infection, we could not perform focus-group interviews or collect data from on-site observations. Fourth, this study was performed over a relatively short period. Future research could examine the psychological experiences of junior nurses over a longer period.

## 5. Conclusions

This research employed a phenomenological approach to generate detailed insights into the transformational experiences of junior nurses who provided direct care to patients with confirmed COVID-19 in an acute care setting. In this study, the hurdles experienced by junior nurses included fear and anxiety, which may be accompanied by somatic symptoms (such as sleep disturbance). A reciprocal pattern of concern (both from the family to the participants and vice versa) was identified in our study. Social media that conveyed negative messages (for example, the death of patients with confirmed COVID-19) may have contributed to the development of negative emotions (such as anxiety) among junior nurses. Our findings also revealed that junior nurses maintained positivity through appreciation of the important relationships in their lives and reflection upon professionalism. The process of self-transformation began when junior nurses were able to confront the hurdles in the early stage of their work in isolation wards with self-care coping strategies. Self-transformation prepared junior nurses to be resilient for future stressful events in the battle against this global pandemic. The confidence of junior nurses built up gradually during self-transformation. Ultimately, junior nurses were more capable of understanding both the negativity and positivity of their experiences. The self-transformation process also enabled junior nurses to recognise and appreciate the wider support system from various parties in society.

## Figures and Tables

**Table 1:** Participant characteristics.

Characteristic		All		Male		Female	
		*n* = 40		*n* = 6		*n* = 34	
		*n*	%	*n*	%	*n*	%
Age							
	21–25	1	2.5	0	0	1	3
	26–30	37	92.5	5	83	32	94
	31–35	2	5	1	17	1	3
Education level							
	Higher diploma	2	5	0	0	2	6
	Bachelor’s degree	38	95	6	100	32	94
Marriage and children							
	Unmarried without children	38	95	6	100	32	94
	Married without children	1	2.5	0	0	1	3
	Married with children	1	2.5	0	0	1	3
Number of year(s) of post-licensure practical experience at time of interview							
	1	0	0	0	0	0	0
	2	6	15	0	0	6	18
	3	18	45	4	67	14	41
	4	16	40	2	33	14	41
Number of months providing direct care to hospitalised patients confirmed with COVID-19 at time of interview							
	6	8	20	0	0	8	24
	7	12	30	2	33	10	29
	8	10	25	1	17	9	26
	9	10	25	3	50	7	21
Religious beliefs							
	None	7	17.5	3	50	4	12
	Christian	10	25	2	33	8	23.5
	Buddhist	23	57.5	1	17	22	64.5
	Other	0	0	0	0	0	0

**Table 2:** Interview guide.

No.	Probing Questions
1.	How do you feel when you are called upon to provide direct nursing care to patients with confirmed COVID-19?
2.	How did your feelings change over time when you practiced as a junior nurse in an isolation ward to take care of these patients day and night?
3.	Can you describe some of your self-care coping strategies to meet your own psychological needs?
4.	In what ways do you think your self-care coping strategies help you on a psychological level?
5.	How do you stay positive when you feel distressed at work?
6.	Reflecting on your months of efforts in caring for patients with COVID-19, how do you perceive the negativity and positivity along the journey?

**Table 3:** Summary of strategies applied to achieve rigor.

Rigor Criteria	Purpose	Strategies to Achieve Rigor	Notes of Application
Credibility	To establish confidence that the results are true, credible, and believable	- Prolonged and varied engagement with each setting	- Engagement through participant observation in the field was infeasible at the time of this research. There was a strict “no third-party visit” policy (as imposed by the Hospital Authority) for the isolation wards admitting patients with confirmed COVID-19. This policy was to prevent the spread of the virus to the community. - However, a range of participants from different acute-care venues were approached through the nursing administration departments that have regular communications about clinical research with the authors’ affiliated institution.
		- Interviewing process and techniques	- The interview guide was tested at two induction meetings after the ethics approval was granted. Two pilot interviews via Zoom were conducted, and the data from these two interviews were also included in the final data analysis.
		- Establishing investigators’ authority	- All members of the research team had the required knowledge, data management skills, and practical experience of no less than 5 years in qualitative research to perform their roles.
		- Collection of referential adequacy materials	- Field notes were used to aid the documentation of the contextual information mentioned by the participants for accurate data analysis. Field notes were also analysed with the transcripts.
		- Peer debriefing	- Debriefing sessions were held regularly at 3-week intervals with the Fellows from the Hong Kong Academy of Nursing to ensure that there were no taken-for-granted biases, perspectives, or assumptions on the researchers’ part.
Dependability	To ensure the findings of this qualitative inquiry would be repeatable if the inquiry occurred within the same cohort	- Rich description of study methods	- The study methods were described in detail and clearly in the research papers.
		- Establishing an audit trail	- All researchers formed a detailed track record of the data-collection process. - Member checking was performed to ensure the clarity of the meanings derived from the participants, thereby enhancing the validity of the accounts from the participants.
		- Stepwise replication of data	- All researchers assessed coding accuracy and inter-coder reliability throughout the data-analysis process.
Confirmability	To extend the confidence that the results would be confirmed/corroborated by other researchers	- Reflexivity	- Measures such as reflexive journals and weekly investigator meetings were adopted.
Transferability	To extend the degree to which the results can be generalised/transferred to other contexts/settings	- Data saturation - Thick description	- Data saturation was reached when no new themes emerged from the participants. All three researchers reached a consensus on the attainment of data saturation. - Lengthy description was provided in the quotes of the participants, such that the meanings of the statements from the participants could be interpreted in context.

## Data Availability

The interview guide is provided in a table in the manuscript. To protect participants’ privacy, the transcripts containing private and confidential data, such as the wards and the sites of practice of the participants, will not be made publicly available.
